# Reliability of point-of-care ultrasound to evaluate fluid tolerance performed by critical care residents

**DOI:** 10.1186/s40001-023-01397-9

**Published:** 2023-10-12

**Authors:** Manuel A. Guerrero-Gutiérrez, Francisco Javier García-Guillén, Humberto Adame-Encarnación, Fernando Monera-Martínez, Silvio A. Ñamendys-Silva, Bertha M. Córdova-Sánchez

**Affiliations:** 1 Instituto Nacional de Cancerología, San Fernando #22, Tlalpan, Mexico City, Mexico; 2 Instituto Nacional de Ciencias Medicas y de la Nutricion Salvador Zubiran, Mexico City, Mexico; 3 Hospital Medica Sur, Mexico City, Mexico

**Keywords:** Agreement, Inferior vena cava, Jugular vein, Time velocity integral, Venous excess ultrasound

## Abstract

**Background:**

Patients with hypotension usually receive intravenous fluids, but only 50% will respond to fluid administration. We aimed to assess the intra and interobserver agreement to evaluate fluid tolerance through diverse ultrasonographic methods.

**Methods:**

We prospectively included critically ill patients on mechanical ventilation. One trained intensivist and two intensive care residents obtained the left ventricular outflow tract velocity–time integral (VTI) variability, inferior vena cava (IVC) distensibility index, internal jugular vein (IJV) distensibility index, and each component of the venous excess ultrasound (VExUS) system. We obtained the intraclass correlation coefficient (ICC) and Gwet’s first-order agreement coefficient (AC1), as appropriate.

**Results:**

We included 32 patients. In-training observers were unable to assess the VTI-variability in two patients. The interobserver agreement was moderate to evaluate the IJV-distensibility index (AC1 0.54, CI 95% 0.29–0.80), fair to evaluate VTI-variability (AC1 0.39, CI 95% 0.12–0.66), and absent to evaluate the IVC-distensibility index (AC1 0.19, CI 95% − 0.07 to 0.44). To classify patients according to their VExUS grade, the intraobserver agreement was good, and the interobserver agreement was moderate (AC1 0.52, CI 95% 0.34–0.69).

**Conclusions:**

Point-of-care ultrasound is frequently used to support decision-making in fluid management. However, we observed that the VTI variability and IVC-distensibility index might require further training of the ultrasound operators to be clinically useful. Our findings suggest that the IJV-distensibility index and the VExUS system have acceptable reproducibility among in-training observers.

## Background

Patients with hypotension usually receive intravenous fluids to increase preload. However, only 50% will be fluid responders by increasing their stroke volume (SV) and cardiac output (CO) [1]. Moreover, excessive fluid administration is associated with mortality [2]. Therefore, determining fluid tolerance, which is the moment a patient can tolerate the administration of fluids without causation of organ dysfunction, helps to avoid excessive fluid administration [3].

Positive-pressure ventilation induces cycling changes in the load of the left and right ventricles. These cyclic changes are greater in fluid responders. Thus, SV variability is a good predictor of fluid responsiveness [4].

A transthoracic echocardiogram allows us to obtain the cross-sectional area (CSA) of the left ventricular outflow tract (LVOT) and the velocity–time integral (VTI) to estimate the SV (SV = CSA × VTI) [5]. Since the CSA is constant during the cardiac cycle, VTI variability also predicts fluid responsiveness [5, 6]. When performed by experienced echocardiographers, the VTI has a good interrater agreement [7]. However, when VTI is measured by medical students after a short training session, its reproducibility decreases [8].

Likewise, ultrasound allows for evaluating inferior vena cava (IVC) diameter changes during the respiratory cycle to predict fluid responsiveness. The IVC distensibility index (100 × [maximum diameter-minimum diameter/minimum diameter]) is widely used. However, its ability to predict fluid responsiveness varies across studies [9, 10]. Most studies evaluating the reproducibility of IVC measurements have been performed in the emergency department and have shown variable interrater agreement [11, 12].

The internal jugular vein (IJV) distensibility index has been evaluated as a predictor of fluid responsiveness [13, 14]. However, there are no studies evaluating the IJV distensibility index interrater agreement.

In addition to cardiac output, organ perfusion depends on the arteriovenous pressure gradient [15]. Therefore, venous congestion due to fluid overload may impair organ perfusion. To evaluate venous congestion through ultrasound, the recently developed Venous Excess Ultrasound (VExUS) score incorporates the IVC diameter and venous Doppler waveform of the portal, hepatic, and interlobular renal veins. The VExUS score has been shown to predict acute kidney injury following cardiac surgery [16]. Currently, there are no studies that evaluated interrater agreement in critically ill patients.

## Methods

### Aim, design, and setting

This study aimed to assess the reliability of point-of-care ultrasound to evaluate fluid tolerance through diverse methods performed by observers with different levels of training. This prospective study was performed in an academic cancer center.

### Participants

We included consecutive patients admitted to the intensive care unit (ICU) from November 2020 to July 2021. Eligible patients were above 18 years old and required mechanical ventilation. Patients with atrial fibrillation or mechanical ventilation in a prone position were excluded.

### Procedures

After inclusion, we registered demographic data, comorbidities, body mass index (BMI), Sequential Organ Failure Assessment (SOFA) score, Richmond Agitation–Sedation Scale, and vital signs. Ultrasonographic evaluations were performed with SonoSite M-Turbo equipment.

All ultrasonographic assessments were performed by three observers. Each observer performed the first assessment. The observers were blinded to each other’s results.

To assess the intraobserver agreement, once the three observers have finished, they performed a second assessment. This measure could have a potential recall bias that must be considered in the interpretation of the results. However, the short time between the first and the second observation prevents the influence of changes due to the clinical condition of critically ill patients.

Observer 1 was a graduate intensivist with postgraduate training in critical care ultrasound and five years of experience. Observers 2 and 3 were intensive care residents with at least three months of training. The current residence program comprises theoretical–practical training for four weeks, followed by a period in which they perform at least thirty ultrasonographic volume assessments under supervision by a graduate intensivist for 2 to 3 weeks. Conventionally, after this time it is considered they can incorporate ultrasonographic fluid assessment into their clinical practice as residents.

During the protocol assessment, patients were placed in a supine position with the head at 30°, mechanical ventilation in mandatory mode, with plateau pressure less than 30 cmH2O, peripheral oxygen saturation (SpO2) more than 95%, and end-expiratory pressure (PEEP) less than 10 cmH2O.

### Left ventricular outflow tract velocity–time integral assessment

The LVOT VTI was evaluated with a sectorial transducer. The probe was placed in the apical position, in the five-chamber view, applying pulsed-wave Doppler at the level of the LVOT. Maximum and minimum values were obtained over five cardiac cycles. LVOT VTI variability was calculated as follows: 100 × [(maximum VTI − minimum VTI)/ (maximum VTI + minimum VTI)/2]. A value of 15% or more was considered a predictor of fluid responsiveness [7].

### Inferior vena cava assessment

The IVC diameter was obtained with a 5-MHz convex transducer. The probe was placed in the subxiphoid position to obtain a long-axis view. The IVC diameter was evaluated during a respiratory cycle. The IVC distensibility index was estimated with the formula [(maximum diameter − minimum diameter)/minimum diameter] × 100. An IVC distensibility index equal to or greater than 18% was considered a predictor of fluid responsiveness [9].

### Jugular vein assessment

The IJV was evaluated with a 10-MHz linear transducer. The IJV was located with two-dimensional ultrasound at the level of the cricoid cartilage. The IJV was identified by compression and color Doppler. We measured the anteroposterior diameter with M-mode ultrasound during a respiratory cycle. The IJV distensibility index was estimated with the formula [(maximum diameter − minimum diameter)/minimum diameter] × 100. An IJV distensibility index equal to or greater than 18% was considered a predictor of fluid responsiveness [13].

### Venous excess ultrasound grading system assessment

The components of the VExUS protocol were evaluated with a 5-MHz convex transducer. To obtain the IVC long-axis view, we placed the probe in the subxiphoid position and obtained the IVC maximum diameter. The original protocol indicates that only in the case of an IVC diameter equal to or greater than 2 cm, the hepatic, portal, and renal veins should be evaluated. However, our study aimed to evaluate the reliability of the measurements; therefore, we performed a complete evaluation independently of the IVC diameter.

We obtained a two-dimensional image of the hepatic veins and applied color flow Doppler and pulse-wave Doppler to assess the diastolic and systolic waveforms. Then, we placed the probe in the right midaxillary line to obtain a two-dimensional image of the right portal vein and initiated pulse-wave Doppler. Then we calculated the pulsatility index with the formula: (maximum velocity-minimum velocity)/maximum velocity. We placed the probe in the posterior axillary line, applied color flow Doppler to find the intrarenal lobar vessels, and used pulse-wave Doppler to evaluate the waveform.

Observers evaluated and classified IVC into two categories according to its diameter: < 2 cm and ≥ 2 cm; hepatic vein in three categories: normal (systolic wave greater than diastolic), mild hepatic vein abnormality (systolic wave smaller than diastolic), and severe hepatic vein abnormality (systolic wave reversal); portal vein in three categories: normal (< 30% pulsatility index), mild portal vein abnormality (30–49% pulsatility index), and severe portal vein abnormality (> 50% pulsatility index); and renal vein in three categories: normal (continuous monophasic flow), mild intrarenal vein abnormality (discontinuous biphasic flow), and severe intrarenal vein abnormality (discontinuous monophasic flow). Observers also assigned a VExUS score as follows: Grade 0 (no congestion): IVC < 2 cm, Grade 1 (mild congestion): IVC ≥ 2 cm and any combination of normal or mildly abnormal patterns, Grade 2 (moderate congestion): IVC ≥ 2 cm and one severely abnormal pattern, Grade 3 (severe congestion) IVC ≥ 2 cm and two or more severely abnormal patterns [15, 16].

We evaluated the agreement to categorize each VEXUS component and to obtain the total VEXUS score.

### Statistical analysis

Considering a Type I error of 0.05, a Type II error of 0.20, and an expected correlation coefficient of 0.6 among observers, we estimated a minimum sample size of 19 patients. Numerical variables are shown as the median and interquartile range (IQR), and categorical variables are shown as proportions (%). We obtained the intraclass correlation coefficient for numerical variables and Gwet’s first-order agreement coefficient (AC1) with the percentage of agreement for categorical variables. We used Altman´s scale to evaluate the strength of agreement [17]. Analyses were processed by STATA 14.0.

## Results

We included 32 patients; their main characteristics are shown in Table 1.

**Table 1:** Clinical characteristics

Variables	Total (*n* = 32)
Male, *n* (%)	14 (43.8)
Age in years, median (IQR)	51 (40–62)
Body mass index kg/m^2^, median (IQR)	27 (21–30)
Body mass index ≥ 30 kg/m^2^, *n* (%)	8 (25)
Diabetes, *n* (%)	4 (12.5)
Hypertension, *n* (%)	8 (25.0)
Systolic pressure mmHg, median (IQR)	108 (99–116)
Diastolic pressure mmHg, median (IQR)	63 (56–72)
Mean arterial pressure mmHg, median (IQR)	79 (71–84)
Central venous pressure mmHg, median (IQR)	12 (8–14)
Heart rate, beats per minute, median (IQR)	92 (78–111)
Positive end-expiratory pressure mmH_2_O, median (IQR)	6 (5–7)
SOFA score, median (IQR)	7 (4–9)
Richmond Agitation–Sedation Scale, median (IQR)	− 5 (− 5 to − 4)
ICU length of stay, days, median (IQR)	6 (3–12)
Hospital length of stay, days, median (IQR)	13 (7–19)
In-hospital death, *n* (%)	14 (43.8)

*IQR* interquartile range, *SOFA* Sequential Organ Failure Assessment, *ICU* intensive care unit

The most experienced observer obtained all of the ultrasonographic measurements, but the in-training observers were unable to assess the LVOT VTI for two patients. The remaining ultrasonographic parameters were obtained in all cases by the three observers (Table 2).

**Table 2:** Obtained values during the first evaluation

Variable	Observer 1	Observer 2	Observer 3
*VTI variables*			
Obtained measurement	32 (100%)	30 (93.8%)	30 (93.8%)
VTI variability	22.6 (18.7–28.3)	20.2 (14.5–29.9)	13.3 (10.3–21.2)
VTI variability ≥ 15%	28 (87.5%)	22 (73.3%)	12 (40.0%)
*IVC variables*			
Obtained measurement	32 (100%)	32 (100%)	32 (100%)
IVC distensibility index	11.4 (7.7–23.7)	17.3 (12.5–32.7)	10.8 (6.3–24.2)
IVC distensibility index ≥ 18%	10 (31.2%)	16 (50.0%)	9 (28.1%)
*IJV variables*			
Obtained measurement	32 (100%)	32 (100%)	32 (100%)
IJV distensibility index	26.8 (12.1–41.8)	42.6 (27.1–80.8)	30.2 (9.2–64.4)
IJV distensibility index ≥ 18%	22 (68.8%)	30 (93.8%)	21 (65.6%)
*VExUS grade*			
Obtained measurement	32 (100%)	32 (100%)	32 (100%)
Grade 0	20 (62.5%)	17 (53.1%)	14 (43.8%)
Grade 1	10 (31.2%)	14 (43.8%)	17 (53.1%)
Grade 2	2 (6.2%)	1 (3.1%)	1 (3.1%)
Grade 3	0 (0.0%)	0 (0.0%)	0 (0.0%)

*IJV* interior jugular vein, *IVC* inferior vena cava, *VTI* velocity–time integral, *VExUS* venous excess ultrasound

Considering the obtained values during the first assessment performed by the three observers, patients were classified as potentially fluid responders in 66.3% (61/92) of VTI evaluations, 76% (73/96) of IJV evaluations, and 36.5% (35/96) of IVC evaluations, and this difference among predictors was statistically significant (*p* < 0.001). According to the VExUS system, observers reported the absence of venous congestion in 53% (51/96) of the evaluations.

Observers showed poor agreement in obtaining the same numerical value of the VTI-variability, IVC-distensibility index, and IJV-distensibility index. However, when these measurements were categorized, according to their cut-off points to predict fluid responsiveness, we observed better agreement among the observers (Table 3).

**Table 3:** Intraobserver and interobserver agreement

	Intraobserver 1		Intraobserver 2		Intraobserver 3		Interobserver	
*VTI variables*								
Variability, ICC (CI95%)	0.04 (− 0.31 to 0.39)	0.405	0.18 (− 0.21 to 0.50)	0.180	0.08 (− 0.27 to 0.42)	0.327	− 0.02 (− 0.20 to 0.22)	0.574
Variability > 15%, AC1 (CI95%)	0.38 (0.01–0.75)	0.044	0.53 (0.20–0.86)	< 0.003	0.01 (− 0.38 to 0.38)	0.995	0.21 (− 0.05 to 0.47)	0.114
Non-responder, % of agreement (CI95%)	0.58 (0.39–0.77)	< 0.001	0.70 (0.52–0.88)	< 0.001	0.50 (0.34–0.73)	< 0.001	0.56 (0.43–0.69)	< 0.001
*IVC variables*								
Distensibility index, ICC (CI95%)	0.08 (− 0.28 to 0.41)	0.338	0.18 (− 0.16 to 0.49)	0.148	0.21 (− 0.13 to 0.51)	0.115	0.13 (− 0.07 to 0.37)	0.105
Distensibility > 18%, AC1 (CI95%)	0.41 (0.07–0.76)	0.021	− 0.04 (− 0.42 to 0.34)	0.845	0.41 (0.06–0.76)	0.021	0.19 (− 0.07 to 0.44)	0.144
Non-responder, % of agreement (CI95%)	0.69 (0.52–0.86)	< 0.001	0.45 (0.29–0.65)	< 0.001	0.69 (0.52–0.86)	< 0.001	0.56 (0.45–0.68)	< 0.001
*IJV variables*								
Distensibility index, ICC (CI95%)	0.10 (− 0.34 to 0.35)	0.481	0.35 (− 0.01 to 0.62)	0.027	0.15 (− 0.21 to 0.47)	0.207	− 0.01 (− 0.17 to 0.23)	0.488
Distensibility > 18%, AC1 (CI95%)	0.45 (0.11–0.80)	0.012	0.85 (0.69–1.00)	< 0.001	0.29 (− 0.07 to 0.66)	0.113	0.54 (0.29–0.80)	< 0.001
Non-responder, % of agreement (CI95%)	0.69 (0.52–0.86)	< 0.001	0.88 (0.75–0.99)	< 0.001	0.63 (0.45–0.80)	< 0.001	0.71 (0.59–0.83)	< 0.001
*VExUS variables*								
IVC ≥ 2 cm, AC1 (CI95%)	0.58 (0.28–0.88)	< 0.001	0.69 (0.42–0.95)	< 0.001	0.63 (0.34–0.91)	< 0.001	0.38 (0.13–0.62)	0.004
Hepatic pattern, AC1 (CI95%)	0.88 (0.75–1.00)	< 0.001	0.92 (0.81–1.00)	< 0.001	0.71 (0.51–0.92)	< 0.001	0.51 (0.32–0.69)	< 0.001
Portal pattern, AC1 (CI95%)	0.62 (0.39–0.85)	< 0.001	0.33 (0.06–0.60)	0.017	0.32 (0.05–0.59)	0.022	0.26 (0.06–0.45)	0.010
Renal pattern, AC1 (CI95%)	0.87 (0.73–1.00)	< 0.001	0.77 (0.53–0.99)	< 0.001	0.83 (0.67–0.99)	< 0.001	0.53 (0.36–0.70)	< 0.000
VExUS grade, AC1 (CI95%)	0.71 (0.50–0.91)	< 0.001	0.62 (0.40–0.83)	< 0.001	0.74 (0.54–0.94)	< 0.001	0.52 (0.34–0.69)	< 0.001
VExUS, % of agreement (CI95%)	0.78 (0.63–0.93)	< 0.001	0.69 (0.52–0.86)	< 0.001	0.81 (0.67–0.96)	< 0.001	0.65 (0.52–0.77)	< 0.001

*VTI* velocity–time integral, *ICC* intraclass correlation coefficient, *AC1* first-order agreement coefficient, *CI* confidence interval, *IVC* inferior vena cava, *IJV* internal jugular vein, *VExUS* venous excess ultrasound

The most experienced observer obtained moderate intraobserver agreement to classify patients as fluid responders with IVC, and IJV. However, in-training observers obtained variable intraobserver agreement for the different methods.

To classify patients as fluid responders, the obtained interobserver agreement was moderate to evaluate the IJV-distensibility index, fair for VTI variability, and absent for IVC variability.

To evaluate venous congestion through the VExUS system, observers obtained a good intraobserver agreement and a moderate interobserver agreement. Despite in-training observers showing a fair intraobserver agreement to evaluate the portal pattern, all observers obtained a good to very good intraobserver agreement assessing the remaining components of the VExUS system.

## Discussion

We evaluated the agreement to acquire and interpret ultrasonographic images performed by one intensivist and two critical care residents. Only the most experienced observer was able to achieve the LVOT VTI assessment in all patients. Villavicencio et al. previously reported an inability to obtain adequate LVOT images in half of the patients evaluated by intensivists with only basic critical care echocardiography training [18]. Previous studies have shown that LVOT VTI assessment relies on the observer experience [7, 8]. However, is a useful tool to predict volume response and to estimate SV [5]. Our findings suggest that extensive and focused training is required before incorporating LVOT VTI into clinical assessment.

Although the IVC distensibility index is a widely used method, we observed low reproducibility among in-training observers. Most studies assessing the agreement between experienced and in-training observers to evaluate IVC-distensibility were performed in the emergency department; they included spontaneously breathing subjects and reported poor to moderate interrater agreement [19, 11]. Rollas et al. reported moderate agreement among intensive care specialists and residents, but only seven patients were mechanically ventilated, and none had a BMI over 30 kg/m^2^ [20]. Our lower agreement may be because all of our patients were mechanically ventilated, and 25% had a BMI over 30 kg/m^2^. These variables could alter the accuracy of the IVC measurements [21].

Recently, the IJV has been proposed as a useful tool to predict fluid responsiveness compared to invasive methods [13]. Additionally, a correlation between the IJV measurement and the central venous pressure in spontaneously breathing patients has been demonstrated [22]. However, its correlation with IVC measurement is not clear [14, 22]. To our knowledge, this is the first study evaluating IJV-distensibility agreement among experienced and in-training observers, and we found moderate agreement, which suggests that it could be a potentially useful tool for in-training physicians.

Currently, the evaluation of venous congestion has become more relevant in the clinical setting. The VExUS system is a novel, noninvasive method to assess venous congestion. Interrater agreement to obtain and interpret the VExUS system has not been previously evaluated. Our results suggest that it could be a reproducible method, even among less experienced observers. Even though intrarenal vein assessment could be difficult because of their small size, we observed a good to very good intraobserver agreement and a moderate interobserver agreement. Previous studies have shown absolute intra and interobserver agreement for classifying the three renal vein patterns that we used [23, 24].

Our results support the need to analyze the agreement among in-training observers to identify the most difficult procedures, reinforce their teaching, and ensure the accuracy of the methods before their clinical use.

This is a single-center study, and we only evaluated three observers (one graduate intensivist and two intensive care residents), which limits the generalizability of the results. Despite being beyond the scope of this study, it is worth mentioning that we did not use a gold standard to define fluid responsiveness, which would have made it possible to establish a comparison among the different predictors of fluid responsiveness.

## Conclusions

Point-of-care ultrasound is frequently used to support decision-making in fluid management. However, we observed that the VTI variability and IVC distensibility index might require further training of the ultrasound operator to be clinically useful. Our findings suggest that the IJV-distensibility index and the VExUS system have acceptable reproducibility among in-training observers.

## Data Availability

The datasets generated and/or analyzed during the current study are not publicly available due to confidentiality reasons but are available from the corresponding author upon reasonable request.

## Abbreviations

AC1: Gwet’s first-order agreement coefficient
BMI: Body mass index
CO: Cardiac output
CSA: Cross-sectional area
ICU: Intensive care unit
IJV: Internal jugular vein
IQR: Interquartile range
IVC: Inferior vena cava
LVOT: Left ventricular outflow tract
PEEP: Positive end-expiratory pressure
SOFA: Sequential Organ Failure Assessment
SpO2: Peripheral oxygen saturation
SV: Stroke volume
VExUS: Venous excess ultrasound
VTI: Velocity–time integral

## References

[1] Marik PE, Cavallazzi R (2013). Does the central venous pressure predict fluid responsiveness? An updated meta-analysis and a plea for some common sense. Crit Care Med.

[2] Marik PE, Lemson J (2014). Fluid responsiveness: an evolution of our understanding. Br J Anaesth.

[3] Kattan E, Castro R, Miralles-Aguiar F, Hernández G, Rola P (2022). The emerging concept of fluid tolerance: A position paper. J Crit Care.

[4] Marik PE, Monnet X, Teboul JL (2011). Hemodynamic parameters to guide fluid therapy. Ann Intensive Care.

[5] Blanco P (2020). Rationale for using the velocity-time integral and the minute distance for assessing the stroke volume and cardiac output in point-of-care settings. Ultrasound J.

[6] Wang J, Zhou D, Gao Y, Wu Z, Wang X, Lv C (2020). Effect of VTILVOT variation rate on the assessment of fluid responsiveness in septic shock patients. Medicine (Baltimore).

[7] Bergenzaun L, Gudmundsson P, Öhlin H, Düring J, Ersson A, Ihrman L, Willenheimer R, Chew MS (2011). Assessing left ventricular systolic function in shock: evaluation of echocardiographic parameters in intensive care. Crit Care.

[8] Koster G, Kaufmann T, Hiemstra B, Wiersema R, Vos ME, Dijkhuizen D, Wong A, Scheeren TWL, Hummel YM, Keus F, van der Horst ICC (2020). Feasibility of cardiac output measurements in critically ill patients by medical students. Ultrasound J.

[9] Barbier C, Loubières Y, Schmit C, Hayon J, Ricôme JL, Jardin F, Vieillard-Baron A (2004). Respiratory changes in inferior vena cava diameter are helpful in predicting fluid responsiveness in ventilated septic patients. Intensive Care Med.

[10] Vignon P, Repessé X, Bégot E, Léger J, Jacob C, Bouferrache K, Slama M, Prat G, Vieillard-Baron A (2017). Comparison of echocardiographic indices used to predict fluid responsiveness in ventilated patients. Am J Respir Crit Care Med.

[11] Akkaya A, Yesilaras M, Aksay E, Sever M, Atilla OD (2013). The interrater reliability of ultrasound imaging of the inferior vena cava performed by emergency residents. Am J Emerg Med.

[12] Bowra J, Uwagboe V, Goudie A, Reid C, Gillett M (2015). Interrater agreement between expert and novice in measuring inferior vena cava diameter and collapsibility index. Emerg Med Australas.

[13] Guarracino F, Ferro B, Forfori F, Bertini P, Magliacano L, Pinsky MR (2014). Jugular vein distensibility predicts fluid responsiveness in septic patients. Crit Care.

[14] Kent A, Patil P, Davila V, Bailey JK, Jones C, Evans DC, Boulger CT, Adkins E, Balakrishnan JM, Valiyaveedan S, Galwankar SC, Bahner DP, Stawicki SP (2015). Sonographic evaluation of intravascular volume status: can internal jugular or femoral vein collapsibility be used in the absence of IVC visualization?. Ann Thorac Med..

[15] Rola P, Miralles-Aguiar F, Argaiz E, Beaubien-Souligny W, Haycock K, Karimov T, Dinh VA, Spiegel R (2021). Clinical applications of the venous excess ultrasound (VExUS) score: conceptual review and case series. Ultrasound J.

[16] Beaubien-Souligny W, Rola P, Haycock K, Bouchard J, Lamarche Y, Spiegel R, Denault AY (2020). Quantifying systemic congestion with Point-Of-Care ultrasound: development of the venous excess ultrasound grading system. Ultrasound J.

[17] Altman DG (1990). Practical statistics for medical research.

[18] Villavicencio C, Leache J, Marin J, Oliva I, Rodriguez A, Bodí M, Soni NJ. Basic critical care echocardiography training of intensivists allows reproducible and reliable measurements of cardiac output. Ultrasound J. 2019;11(1):5. 10.1186/s13089-019-0120-0 10.1186/s13089-019-0120-0 PMC6638616 31359188

[19] Fields JM, Lee PA, Jenq KY, Mark DG, Panebianco NL, Dean AJ. The interrater reliability of inferior vena cava ultrasound by bedside clinician sonographers in emergency department patients. Acad Emerg Med. 2011;18(1):98–101. 10.1111/j.1553-2712.2010.00952.x 10.1111/j.1553-2712.2010.00952.x 21414063

[20] Rollas K, Kilicaslan B, Erden A (2021). The interrater reliability of inferior vena cava ultrasonography performed by intensive care fellows. J Crit Intensive Care.

[21] Millington SJ. Ultrasound assessment of the inferior vena cava for fluid responsiveness: easy, fun, but unlikely to be helpful. Can J Anaesth. 2019;66(6):633–8. 10.1007/s12630-019-01357-0. 10.1007/s12630-019-01357-0 30919234

[22] Parenti N, Scalese M, Palazzi C, Agrusta F, Cahill J, Agnelli G (2019). Role of internal jugular vein ultrasound measurements in the assessment of central venous pressure in spontaneously breathing patients: a systematic review. J Acute Med.

[23] Iida N, Seo Y, Sai S (2016). Clinical implications of intrarenal hemodynamic evaluation by doppler ultrasonography in heart failure. JACC Heart Fail.

[24] Nijst P, Martens P, Dupont M, Tang WHW, Mullens W (2017). Intrarenal flow alterations during transition from euvolemia to intravascular volume expansion in heart failure patients. JACC Heart Fail.

